# New Opportunities for Forest Remote Sensing Through Ultra-High-Density Drone Lidar

**DOI:** 10.1007/s10712-019-09529-9

**Published:** 2019-05-04

**Authors:** James R. Kellner, John Armston, Markus Birrer, K. C. Cushman, Laura Duncanson, Christoph Eck, Christoph Falleger, Benedikt Imbach, Kamil Král, Martin Krůček, Jan Trochta, Tomáš Vrška, Carlo Zgraggen

**Affiliations:** 1 Institute at Brown for Environment and Society, Brown University, 85 Waterman Street, Providence, RI 02912 USA; 2 Department of Ecology and Evolutionary Biology, Brown University, 80 Waterman Street, Providence, RI 02912 USA; 3 Department of Geographical Sciences, University of Maryland College Park, 2181 LeFrak Hall, College Park, MD 20740 USA; 4 Aeroscout GmbH, Hengstrain 14, 6280 Hochdorf, Switzerland; 5 The Silva Tarouca Research Institute, Department of Forest Ecology, Lidicka 25/27, 602 00 Brno, Czechia

**Keywords:** Drone, Global Ecosystem Dynamics Investigation (GEDI), Lidar, Remote sensing, UAV

## Abstract

Current and planned space missions will produce aboveground biomass density data products at varying spatial resolution. Calibration and validation of these data products is critically dependent on the existence of field estimates of aboveground biomass and coincident remote sensing data from airborne or terrestrial lidar. There are few places that meet these requirements, and they are mostly in the northern hemisphere and temperate zone. Here we summarize the potential for low-altitude drones to produce new observations in support of mission science. We describe technical requirements for producing high-quality measurements from autonomous platforms and highlight differences among commercially available laser scanners and drone aircraft. We then describe a case study using a heavy-lift autonomous helicopter in a temperate mountain forest in the southern Czech Republic in support of calibration and validation activities for the NASA Global Ecosystem Dynamics Investigation. Low-altitude flight using drones enables the collection of ultra-high-density point clouds using wider laser scan angles than have been possible from traditional airborne platforms. These measurements can be precise and accurate and can achieve measurement densities of thousands of points · m^−2^. Analysis of surface elevation measurements on a heterogeneous target observed 51 days apart indicates that the realized range accuracy is 2.4 cm. The single-date precision is 2.1–4.5 cm. These estimates are net of all processing artifacts and geolocation errors under fully autonomous flight. The 3D model produced by these data can clearly resolve branch and stem structure that is comparable to terrestrial laser scans and can be acquired rapidly over large landscapes at a fraction of the cost of traditional airborne laser scanning.

## Introduction

We are on the cusp of a golden age in satellite remote sensing. Current and forthcoming missions will produce measurements of the land surface with unprecedented spatial and temporal detail. The use of the International Space Station (ISS) as a platform for coordinated Earth observation will generate remote sensing data over diurnal timescales, including characterizations of canopy temperature, solar-induced fluorescence, and ecosystem structure (Stavros et al. 2017). The data products from these missions will enable new insight into the biological underpinnings of the Earth system and help to constrain uncertainties in the global distribution of aboveground stocks and fluxes of carbon and water.

Three missions will quantify aboveground biomass density (AGBD). The Global Ecosystem Dynamics Investigation (GEDI) is producing globally representative measurements of vertical height profiles (waveforms) and estimates of aboveground carbon stocks (Dubayah et al. 2014; Hancock et al. 2019). Two additional efforts are the NASA-ISRO Synthetic Aperture Radar (NISAR) and ESA P-band radar (BIOMASS), both of which are free-flying satellites that will produce AGBD data products (Scipal et al. 2010; NISAR Mission Science Handbook 2018).

Calibration and validation of these data products is critically dependent on the existence of globally representative field estimates of AGBD and coincident remote sensing measurements of vegetation structure (Duncanson et al. 2019). The best sources of remotely sensed vegetation structure are high-density airborne or terrestrial laser scanning (ALS and TLS, respectively), but there are relatively few places with field estimates of AGBD and coincident remote sensing data, and they are disproportionately in the northern hemisphere and temperate zone. In contrast, the tropics contain substantial aboveground forest carbon stocks (Pan et al. 2011), but are not as well represented in existing field inventories used for calibration and validation, especially those of Africa and Asia.

The use of traditional aircraft with lidar instruments to acquire remote sensing measurements of vegetation structure that is coincident with field estimates of AGBD is cost-prohibitive for a large number of remote locations, many of which are far from major centers. It would also be inefficient, because the critical areas necessary for calibration and validation activities are the plots and surrounding landscapes < 10 km^2^ (Held et al. 2015).

### New Opportunities

An alternative to traditional airborne laser scanning is to obtain these data using drones. Lidar from low-altitude drones is fundamentally similar to traditional airborne laser scanning. Sensors record the return-time of emitted laser pulses and combine this information with location and attitude to project recorded laser pulses in a 3D coordinate system (Lefsky et al. 2002). But the methods of data collection from low-altitude flight create new opportunities to characterize the three-dimensional structure of forests in ways that have not been possible until now.

There are several important differences. Low-altitude flight at relatively slow speeds can produce point densities that are orders of magnitude greater than traditional airborne laser scanning. Coupled with wide scan angles, these large point densities can resolve individual tree and branch structure and are similar to TLS. Low-altitude flight reduces the impact of GPS and pointing uncertainties that accumulate with the distance between sensors and the terrain or vegetation surface, making wide-angle scans possible. Because drone flight is more firmly under the control of the investigator and about one order of magnitude less costly than traditional airborne laser scanning, flight plans can be developed to collect high-density measurements in novel ways that enable hypothesis testing, or evaluate the impact of collection scenarios on remote sensing measurements.

**Fig. 1:**
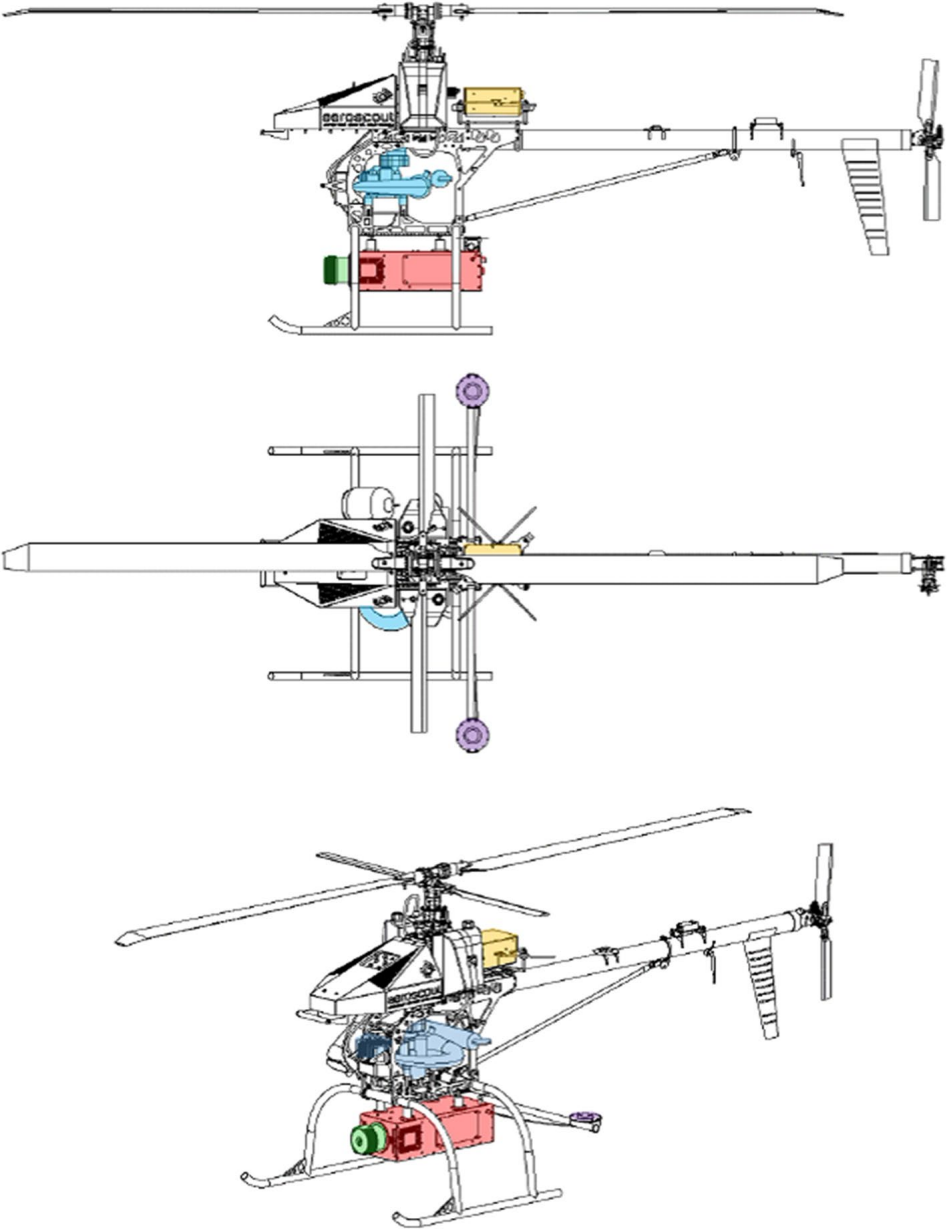
The Aeroscout GmbH B100 heavy-lift autonomous helicopter. Components are distinguished in color: flight control system hardware (orange), engine (blue), dual GPS antennas (purple), RIEGL VUX-1 laser scanner (green), GPS-IMU and electrical supply (red). The main rotor is 3.2 m in length, and the aircraft weighs 77 kg with maximum payload

Below we summarize the potential for drones to produce new observations in support of mission science. In the first section, we describe technical considerations for producing high-quality measurements from autonomous platforms, and we highlight differences among commercially available laser scanners and drone aircraft. We next describe a case study using the Brown Platform for Autonomous Remote Sensing (BPAR, Fig. 1) in a temperate mountain forest in the southern Czech Republic in support of calibration and validation activities for GEDI. We demonstrate that drone measurements can facilitate large-area mapping and the high-quality measurements necessary for validation of mission data products. We conclude by addressing three areas where drone remote sensing is likely to make significant contributions to our understanding of the land surface and to meeting the calibration and validation needs of current and forthcoming space missions.

## Technical Considerations for Drone Remote Sensing

Generating high-quality remote sensing data from an unmanned platform requires a stable aircraft with a robust flight control system, a survey-grade GPS and inertial motion unit (GPS-IMU), vibration isolation of sensor hardware, and post-processing using differential correction. In general, vibration isolation and stability are easier to control in heavier aircraft than lighter ones. Larger aircraft can resist wind and thermal activity, which allows them to operate under a wider range of conditions than smaller aircraft. Long flight durations are necessary to map areas larger than a few ha (Brede et al. 2017).

### Light-Weight Airborne Laser Scanners

In the last few years, a number of commercially available, light-weight laser scanners have been developed that are suitable for drone-based flight operations (Table 1). Some of these sensors are developed by RIEGL Laser Measurement Systems, the Austrian manufacturer of survey-grade lidar instruments. Others were originally designed for automotive or industrial use, but have been repurposed for drone flight. These sensors differ in weight, range accuracy, beam divergence (and therefore footprint size and shape), wavelength, the maximum measurement rate, the number of returns recorded, the effective measurement range (EMR), and scan angle range (Table 1). The RIEGL sensors are heavier, more accurate, produce higher point densities, and are the only lightweight sensors capable of recording > 3 returns per emitted laser pulse. They also have the narrowest beam divergences and therefore result in the smallest footprints at a given flight altitude. For example, the RIEGL VUX-1 has a 0.5 mrad beam divergence, which produces a 5 cm circular footprint at a distance of 100 m. Contrast this with the Velodyne HDL-32, an industrial laser scanner repurposed for drone flight, which has a 3 × 1.5 mrad beam divergence (i.e., the beam divergence is different in the across-beam dimensions). This produces a rectangular footprint of 30 × 15 cm at a distance of 100 m.

**Table 1:** Characteristics of lightweight commercial lidar instruments

Manufacturer	Model	Weight (kg)	Range accuracy (cm)	Beam divergence (mrad)	Laser Wavelength (nm)	Max. measurement rate (kHz)	No. returns	Max. measurement range (m)	FOV (deg.)
RIEGL	VUX-240	3.8	2.0	0.35 × 0.35	1550	1500	Multi	350	75
RIEGL	VUX-1	3.5	1.0	0.5 × 0.5	1550	500	Multi	170	330
RIEGL	VUX-1HA	5.0	0.5	0.5 × 0.5	1550	1000	Multi	120	360
RIEGL	VUX-1LR	3.5	1.5	0.5 × 0.5	1550	750	Multi	215	330
RIEGL	miniVUX-1	1.55	1.5	1.6 × 0.5	905	100	5	150	360
Velodyne	VLP-16	0.83	2.0	3.0 × 1.5	903	600	2	100	360
Velodyne	HDL-32	1.0	2.0	3.0 × 1.5	903	1390	2	100	360
Ibeo	LUX	1.0	4.0	14.0 × 1.4	905	38	3	100	110

Data provided by manufacturers unless otherwise indicated. Maximum measurement range depends on measurement rate and target reflectance. For the RIEGL instruments, the maximum measurement range is at the maximum measurement rate on a 20% reflectance target. For Velodyne and Ibeo instruments, the maximum measurement range is under unspecified conditions. Characteristics for the Ibeo LUX are from Lin et al. (2013)

### Precision and Accuracy

The precision and accuracy of all airborne laser scans is influenced by the stability of the sensor during flight, knowledge of the sensor attitude, typically from a survey-grade GPS-IMU, and flight altitude. Because GPS and pointing errors accumulate with distance from the target, low-altitude flight can partially compensate for GPS-IMU quality, although a poor GPS-IMU can result in data that are not useful (but see Sect. 3.2 on computer vision). Stated differently, the same GPS-IMU will result in better horizontal placement of projected laser pulses at a lower flight altitude than a higher one. For a sensor at altitude $A$, the horizontal position of a projected laser pulse along a single axis can be calculated as:

1 $${\text{Horizontal position}} = A \times \tan \left( {{\text{scan angle}} \pm \epsilon } \right)$$

For the same value of $A$, a given $\epsilon$, which denotes error in the scan angle measurement, has a larger impact on the horizontal position when the scan angle is larger. For a given scan angle and $\epsilon$, error in the horizontal position is proportional to $A$. By operating at low altitude, drone flight minimizes error in horizontal position of projected laser pulses.

The best ranging accuracies reported for laser scanners are from terrestrial sensors, which are on the order of one to a few mm at distances of 100 m (Calders et al. 2017; Disney et al. 2018). No airborne sensors currently approach this degree of performance during flight operations (Table 1).

### Footprint Size

The interpretation of ranging accuracies becomes challenging inside canopies due to differences in beam divergence, distance from the target, and consequently, footprint size. Beam divergence is the rate of increase in beam size with distance from the sensor. Just as a flashlight makes a larger circle on a wall from a greater distance, laser scans produce larger footprints when the sensor is farther away from a target reflective surface. The combination of footprint size, distance from the target, and target reflectivity fundamentally constrains the spatial resolving capacity of any laser scanner (Lichti and Jamtsho 2006; Milenković et al. 2018). Commercial TLS instruments produce footprint sizes in the 2–5 cm range at 100 m (Disney et al. 2018), allowing them to resolve small stems and branches. The RIEGL VUX-1 produces a footprint size of 5 cm at 100 m from the target. Other laser scanners that are being operated from low-altitude drones have variable beam divergences and a wide range of footprint sizes (Table 1). Traditional airborne laser scanners produce footprints in the 15–30 cm range from flight altitudes in the 500–1000 m range. Thus, airborne laser scanners that exhibit similar fundamental ranging accuracies may not be equally amenable to detection of branch and stem structure within canopies. This is because resolving fine structures requires high accuracy, high density, and small footprints (Disney et al. 2018). Because footprint size is a function of beam divergence and distance from the target, laser scanners operated from low-altitude produce footprints that are relatively more variable in size along the beam path. For example, at a flight altitude of 100 m over a 50 m canopy, footprint size for the VUX-1 is 2.5 cm on the canopy top and 5 cm on the ground. This is an absolute difference of 2.5 cm, but the ground footprints are twice as large. At 500 m altitude, the VUX-1 produces 22.5 cm footprints on a 50-m canopy and 25 cm footprints at ground level. The absolute difference is still 2.5 cm, but the ground footprints are now only 11% larger. This indicates that the potential for footprints to contain qualitatively different information along the beam path, such as individual leaf and small branch returns, is greater when the distance to the target is less.

### Effective Measurement Range

EMR is the maximum distance over which a laser scanner can record a reflected laser pulse. The EMR depends on the size and reflectivity of the target and energy per emitted laser pulse (pulse repetition rate, PRR). For example, the EMR of the RIEGL VUX-1 at a PRR of 300 kHz is 230 m for a target of 20% reflectance, or 400 m for a target of 60% reflectance. The wavelength of the VUX-1 is 1550 nm. The reflectance of green vegetation is about 10% at 1550 nm, and woody vegetation has a reflectance of about 30% at 1550 nm (Asner and Heidebrecht 2002). The EMR of the VUX-1 increases to 400 and 660 m for targets of 20% and 60% reflectance, respectively, at a PRR of 100 kHz because there is more energy per emitted laser pulse. Velodyne does not report EMR for targets of varying reflectivity, but the reported EMR is 100 m. Lin et al. (2013) report an EMR for the Ibeo LUX lidar sensor of 100 m. These considerations are important, because the beam path must be < EMR to guarantee that returns can be recorded. At a scan angle of 60°, the beam path is two times the sensor altitude. A good rule of thumb is that flight altitude should not exceed 0.5 × EMR when scan angles up to 60° are employed.

### Drone Platforms

Just as the number of commercially available laser scanners has rapidly increased over the last few years, so too have the number of drone aircraft capable of carrying these instruments. These platforms range in size from battery-powered multirotor aircraft that weigh a few kg and can carry a small laser scanner for a few minutes, like those developed by the company Phoenix Lidar Systems, to heavy-lift gasoline powered helicopters like the Aeroscout GmbH B330 that can carry a 50-kg payload for three hours. Rotor-powered aircraft of intermediate flight duration and payload capacity include the electric powered Vapor 55 helicopter developed by Pulse Aerospace. This traditional helicopter can carry a 5-kg payload for 1 h. The RIEGL RiCOPTER is a multirotor developed by RIEGL Laser Measurement Systems to carry the VUX-line scanners. It has a 6.5-kg payload capacity and a maximum flight duration of 0.5 h. In general, fixed-wing aircraft have longer flight durations than helicopters or multirotor aircraft of the same size. For example, the Edinburgh-based company Carbomap is developing a long-endurance fixed-wing platform that will rival the large-area mapping ability of traditional airborne laser scanning from crewed aircraft. The long flight duration of fixed-wing aircraft comes at the cost of sacrificing control over flight speed.

Combinations of laser scanners, GPS-IMU, and aircraft dictate the capabilities and measurement quality that can be expected from drone-based laser scans. Smaller aircraft and sensors are nimble and can be deployed easily and within the scope of existing regulations. Larger aircraft may require explicit permission from national departments of civil aviation and a professional team of pilots and payload operators. Some aircraft and laser scanners can be subject to export control regulations. Larger aircraft can carry higher-quality laser scanners and GPS-IMU. The selection of an aircraft, laser scanner, and GPS-IMU hardware should therefore consider measurement requirements, including point density, accuracy, the required extent of areal coverage, and flight time.

## Brown Platform for Autonomous Remote Sensing

The Brown Platform for Autonomous Remote Sensing is a suite of sensors carried by a heavy-lift helicopter developed by the Swiss company Aeroscout GmbH. The BPAR sensor package includes imaging spectrometers that cover the 400–2500 nm region of the solar spectrum at varying resolution and sampling intervals, a 24.3 MP digital camera, a RIEGL VUX-1 laser scanner (Table 1), and an Oxford Technical Solutions (OXTS) Survey +2 GPS-IMU. The VUX-1 is a 1550 nm laser capable of recording up to 500,000 measurements s^−1^. The sensor records the return time of emitted laser pulses and processes the recorded waveform to identify discrete reflective surfaces (returns) within every emitted laser pulse. The OXTS GPS-IMU is a survey-grade instrument designed for airborne mapping applications. The nominal roll and pitch accuracy are 0.03°, and the nominal heading accuracy in dual-antenna mode is 0.05°. In our configuration, the instrument records at 250 Hz and has access to the GPS and GLONASS networks. During flight operations, we collect an independent global navigation satellite system (GNSS) data stream on the ground using a Novel FlexPak 6 Triple Frequency + L-band GNSS receiver. This data stream is used to differentially correct the OXTS GPS-IMU measurements in post-processing.

The aircraft and sensors are operated by three people. A certified pilot in command (PIC) is responsible for takeoff and landing under manual control. The PIC has access to a forward-facing video camera that is mounted on the front of the aircraft. The PIC can operate the aircraft using hand controls by line-of-sight or using a video-feed to the forward-facing camera. Upon reaching altitude, the PIC communicates with the ground-station pilot (GSP) by radio. The GSP interacts with the flight control system over an 868 MHz data link that provides information about location, altitude, speed, heading, engine performance, and exhaust characteristics. The GSP is responsible for engaging the flight control system to initiate autonomous flight, after which the aircraft will visit a set of locations in a specified order at a specified altitude and speed under autonomous control. During the flight, a payload operator (PO) communicates with onboard sensors over a 2.4 GHz data link to monitor the data collection in real time. The PO can communicate with the PIC and GSP to request changes to the flight plan when needed. When the mission is complete, the aircraft will return to a specified location and hover, awaiting further instructions from the PIC.

The aircraft used during campaigns in the Czech Republic was the Aeroscout B100, which is operated with rigorous failsafe mechanisms and multiple levels of redundancy. With the exception of the flight crew, no people are within 100 feet (30.5 m) of the aircraft during flight operations, and we design flight plans that minimize risk to people and property. When a road or trail crossing is unavoidable, we cross at a right angle and visually confirm that there are no people beneath the aircraft trajectory. The aircraft has a redundant flight control system and electrical supply. We program instructions into the flight control system so that the aircraft will safely land in the event that communication is severed. If this occurs, the aircraft will hover at a specified location for 10 min, or until remaining fuel falls below a critical level, then land autonomously. The engine is gasoline powered and can support an 18-kg payload for 1.5 h at sea level. The maximum takeoff weight is 77 kg, and the aircraft operates under certificate of the Swiss Civil Aviation Authority (BAZL).

### Flight Campaign in the Czech Republic

We collected airborne laser scans using BPAR over a temperate mountain forest in the southern Czech Republic. The site contains the Zofin Forest Dynamics Plot, which is a 25 ha permanent-inventory plot in which all free-standing woody plants > 1 cm diameter at breast height (DBH) have been mapped and monitored since 2012 (Anderson-Teixeira et al. 2015; Janík et al. 2016). This forest is dominated by old-growth European beech (*Fagus sylvatica*) and Norway spruce (*Picea abies*), with occasional silver fir (*Abies alba*). The surrounding landscape has been actively managed for Norway spruce production and includes planted and clearcut areas.

**Fig. 2:**
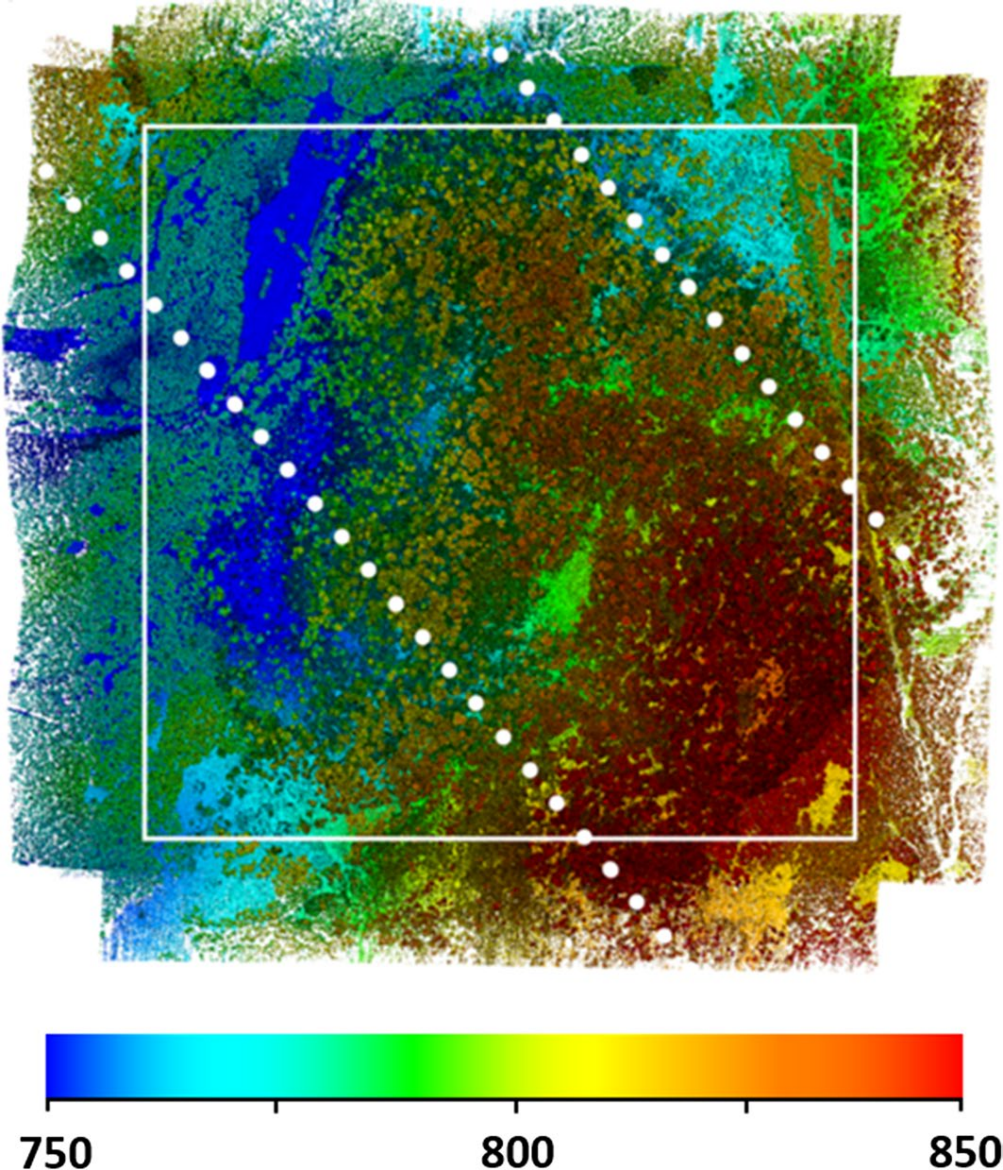
Operational drone lidar in support of mission science. We collected these data using the Brown Platform for Autonomous Remote Sensing in a temperate beech forest in the southern Czech Republic in April, 2018. Colors indicate elevation (m), and the tallest trees are about 40 m. The white box is 1 by 1 km and equal in size to the GEDI L4B aboveground biomass density data product. White dots are simulated GEDI L4A ground tracks, drawn to scale (22 m footprint diameter, 60 m along-track spacing, 600 m across-track spacing, track inclination is arbitrary). These data were collected in five flight hours using 90 flight lines at 90° angles. Mean point density is 2801 points · m^−2^. Collecting lidar data at 100 points · m^−2^ takes about 6 min · km^−2^ using this platform

The data were collected in two sets of orthogonal flight lines that we repeated 51 days apart. The first flights commenced on April 16, 2018. The second flights started on June 6, 2018. These dates were selected to produce observations under leaf-off and leaf-on conditions with little to no change in woody structure. The April campaign was completed in six flights over two consecutive days. The June campaign required six flights over three consecutive days. The total flight time for each campaign was about 5 h. For each campaign, there were 45 flight lines in the NW–SE direction and 45 flight lines in the NE-SW direction. The purpose of using two sets of flight lines over the same area was to ensure dense point coverage of stem and branch structure. The nominal flight altitude was 110 m aboveground, and the nominal flight speed was 6 m · s^−1^. During the autonomous portion of the flight, the flight control system maintained stable control of the aircraft and sensors. For example, during a representative flight line the realized speed was 6 m · s^−1^ (standard deviation, SD = 0.06 m s^−1^). The standard deviation in the pitch, roll, and heading axes was 0.3°, 0.6°, and 0.8°, respectively. The total areas covered were 1.72 and 1.60 km^2^, respectively (Fig. 2).

Measurement density is large. The leaf-off point cloud contains 4,811,370,798 unique returns, corresponding to a mean density of 2801 · m^−2^. The leaf-on point cloud, acquired over the same location 51 days later, contains 3,465,943,773 unique returns. This is a mean density of 2166 · m^−2^. Within the primary area of interest, point density exceeds the landscape mean in forested areas, because the entire landscape includes edges with reduced overlap among flight lines, and open forest with fewer higher-order returns (Table 2). For example, a representative 2.25 ha area dominated by old-growth *F. sylvatica* contains 97,615,242 points under leaf-off conditions, corresponding to a density of 4438 · m^−2^, and 67,769,379 points under leaf-on conditions. The leaf-on density is 3012 · m^−2^.

**Table 2:** The distribution of return numbers (percentages) from airborne laser scans using a low-altitude drone under leaf-off and leaf-on conditions 51 days apart

	First	Second	Third	Fourth	≥ Fifth
Closed forest leaf off	43.0	31.1	17.2	6.8	1.9
Closed forest leaf on	63.7	27.8	7.1	1.2	0.2
Open forest leaf off	83.2	13.6	2.8	0.4	0.0
Open forest leaf on	86.8	11.5	1.5	0.2	0.0

The measurements were collected using the Brown Platform for Autonomous Remote Sensing and contrast a closed-canopy old-growth forest dominated by European beech (*F. sylvatica*) with an open area dominated by Norway spruce (*P. abies*) and silver fir (*A. alba*). Higher-order returns occur more frequently in closed-canopy forest due to complex vertical structure. The presence of leaf area reduces return numbers > 3

### Precision and Accuracy of Airborne Laser Scans Under Autonomous Flight

**Fig. 3:**
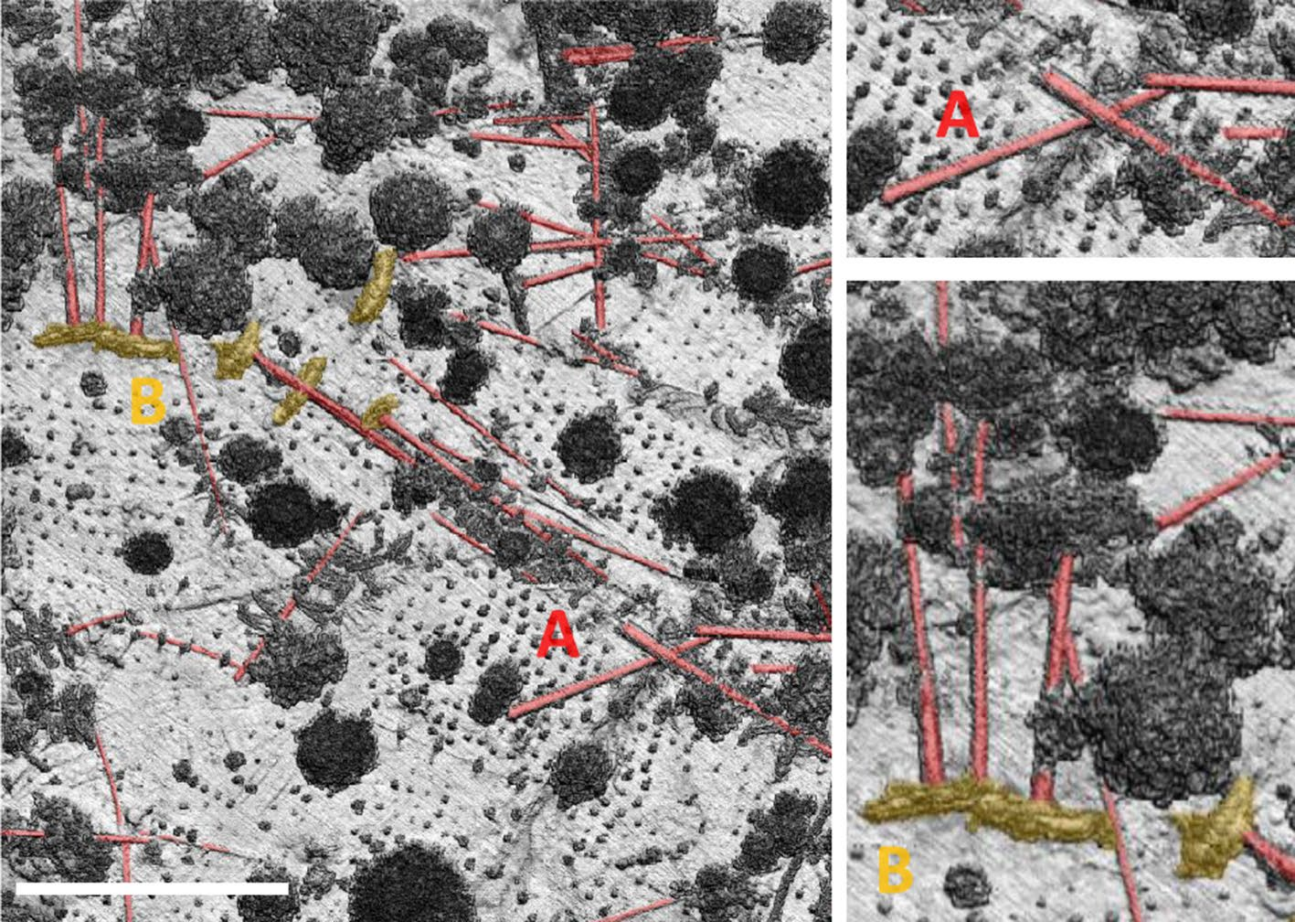
A high-density digital surface model from drone lidar resolves recently fallen trees. The image is a 5 cm digital surface model and colors indicate sun-shaded intensity. Fallen trees are labeled in red. Tip-up mounds are orange. The white scale bar in the main image is 25 m

**Fig. 4:**
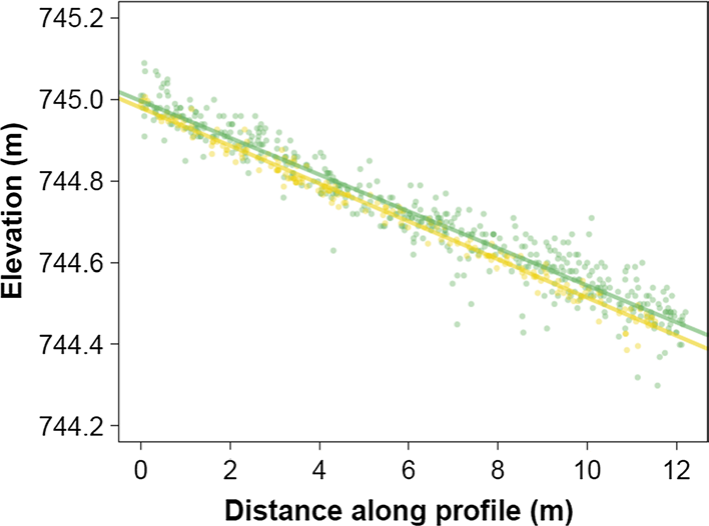
Comparison of lidar surface elevation profiles from April 16, 2018 (green points and line) and June 6, 2018 (orange points and line). The profile is 2 cm wide and was extracted from the stem of a fallen tree (labeled A in Fig. 3). The mean difference in elevation between these lines is 2.4 cm. The residual standard error is 4.5 cm in April and 2.1 cm in June

We quantified range precision and accuracy for the RIEGL VUX-1 during autonomous flight by extracting points along a 12 m profile on the stem of a fallen dead tree that was apparent in the high-density point cloud (Figs. 3, 4). Linear regressions under leaf-off and leaf-on conditions had very high coefficients of determination and similar slope and intercept terms (leaf off: *b*0 = 745.0 m, *b*1 = − 4.51 cm, *r*^2^ = 0.930, residual standard error (RSE) = 4.51 cm; leaf on: *b*0 = 745.0 m, *b*1 = − 4.65 cm, *r*^2^ = 0.984, RSE = 2.05 cm). The mean difference between the predicted values is 2.4 cm, and ranges from 3.2 to 1.7 cm. From this analysis, we conclude that the realized ranging accuracy in the post-processed point cloud has a standard deviation < 5 cm. We emphasize that this is the ranging accuracy net of all processing artifacts and geolocation errors under fully autonomous flight on a heterogeneous target (the fallen dead tree) and that this number is about one order of magnitude greater (worse) than the ranging accuracies reported for TLS (Calders et al. 2017; Disney et al. 2018). It is of a similar or smaller magnitude to ranging accuracies reported for traditional airborne laser scanning (Asner et al. 2007; Kellner et al. 2009).

Recent developments in computer vision may be able to improve the precision and accuracy of airborne laser scans, potentially compensating for low-quality GPS-IMU measurements deployed on aircraft with limited payload capacity and increasing the performance of survey-grade instruments. Simultaneous localization and mapping (SLAM) algorithms are used to facilitate autonomous navigation in unstructured environments lacking GPS (Aouf et al. 2009; Qiang and Xin-sheng 2013). These methods work on 2D video streams from moving vehicles to solve the 3D geometry of the local environment. Although normally used for real-time navigation, this information provides a trajectory independent of the GPS-IMU that can constrain the position and pointing angles of the sensor. In a pioneering study, Wallace et al. (2012) collected airborne lidar from a multirotor aircraft using an Ibeo LUX laser scanner and low-quality GPS-IMU (Table 1). The horizontal RMSE was 0.61 m when data were processed using only the GPS-IMU. However, a high-definition video stream was collected at 30 frames per second during flight and used to produce an independent trajectory by applying the scale-invariant feature transform (SIFT) algorithm to the sequence of 2D images (Lowe 1999). The authors integrated the GPS-IMU and computer vision trajectories using a Kalman filter. This reduced the horizontal RMSE to 0.34 m. Thus, post-processing using computer vision approximately halved the horizontal uncertainty. No study has systematically evaluated the potential of this approach for improving the horizontal accuracy of airborne laser scans, but doing so is a high research priority.

## Promising Applications of Drone Remote Sensing

### Individual Tree Segmentation

**Fig. 5:**
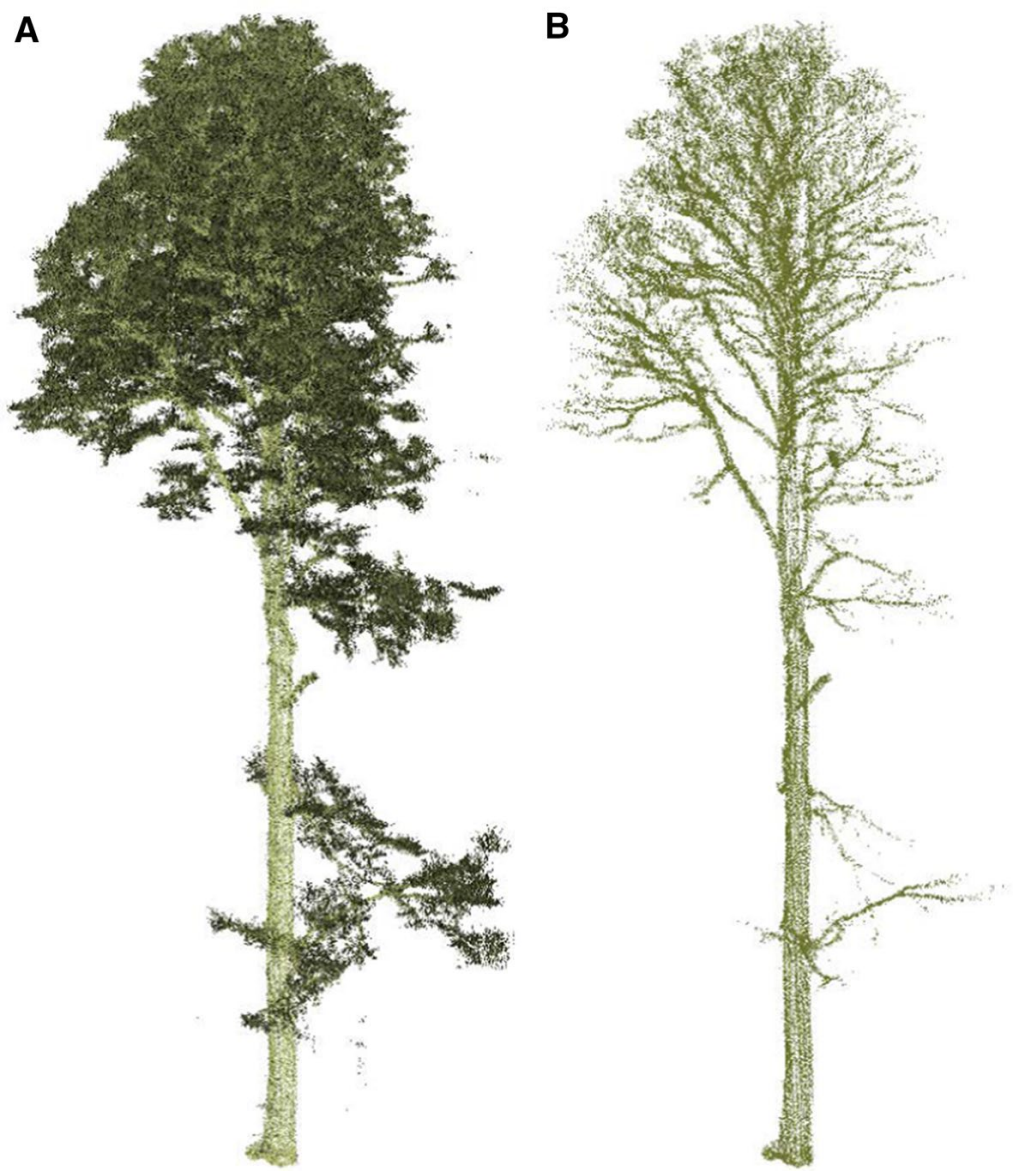
A single European beech (*F. sylvatica*) in the high-density point cloud acquired by the Brown Platform for Autonomous Remote Sensing. **a** All returns. **b** Only returns with 16-bit reflectance intensity > 45,000. The intensity filter removes leaf material and shows branch and stem structure necessary for automated segmentation

One of the most promising applications of drone remote sensing is the segmentation of individual stems using methods originally developed for terrestrial laser scanning (Fig. 5). Applying these methods to ultra-high-density point clouds that contain millions of trees is a formidable computational problem (Raumonen et al. 2013). Overcoming this challenge would beat back a series of pervasive limitations to quantifying AGBD using remote sensing and create new opportunities for demographic analysis of tree populations using much larger samples than are currently available (Kellner and Hubbell 2017). The essential problem is that no remote sensing method or conventional forest inventory directly measures mass. The most commonly measured variables are stem diameter and height. These quantities are used to predict AGB using allometric scaling equations, which are themselves developed from a relatively small number of trees that have been measured, harvested, and weighed (Jenkins et al. 2003; Muukkonen 2007; Chave et al. 2014). The samples used to build these scaling equations are nonrandom and almost certainly biased in favor of trees with idealized forms (Clark and Kellner 2012).

The only way to evaluate the accuracy of these scaling equations is to compare predicted to measured AGB. The few studies that have undertaken this arduous task have shown that the equations are biased, especially among the largest trees (Calders et al. 2015; Gonzalez de Tanago et al. 2018). One way to lessen the impact of this problem is to compute wood volume using very high density laser scans (Raumonen et al. 2013; Calders et al. 2015; Gonzalez de Tanago et al. 2018). Multiplying wood volume by wood density results in an estimate of AGB that is independent of the allometries. Quantitative structure models (QSMs) segment individual trees within high-density point clouds and fit large numbers of cylinders to individual tree objects (Raumonen et al. 2013; Åkerblom et al. 2015). The accumulated volume of these cylinders is an estimate of tree-level wood volume. Three studies have compared these numbers to harvested trees. They found that wood volume and AGB quantified using QSMs are unbiased (Calders et al. 2015; Gonzalez de Tanago et al. 2018; Takoudjou et al. 2018).

Automated segmentation methods therefore hold great promise, because they will improve the quality of existing scaling equations and are likely to eventually replace them. The most important limitation to this approach is that it remains dependent on intensively collected field data from TLS and is therefore difficult to apply throughout large areas needed for the calibration and validation of data products from current and forthcoming space missions. For example, the GEDI L4B gridded AGBD data product has a resolution of 1 km^2^ (Patterson et al. 2019). Low-altitude drone flight may be able to meet this challenge by producing high-density point clouds throughout areas of 1–10 km^2^.

**Fig. 6:**
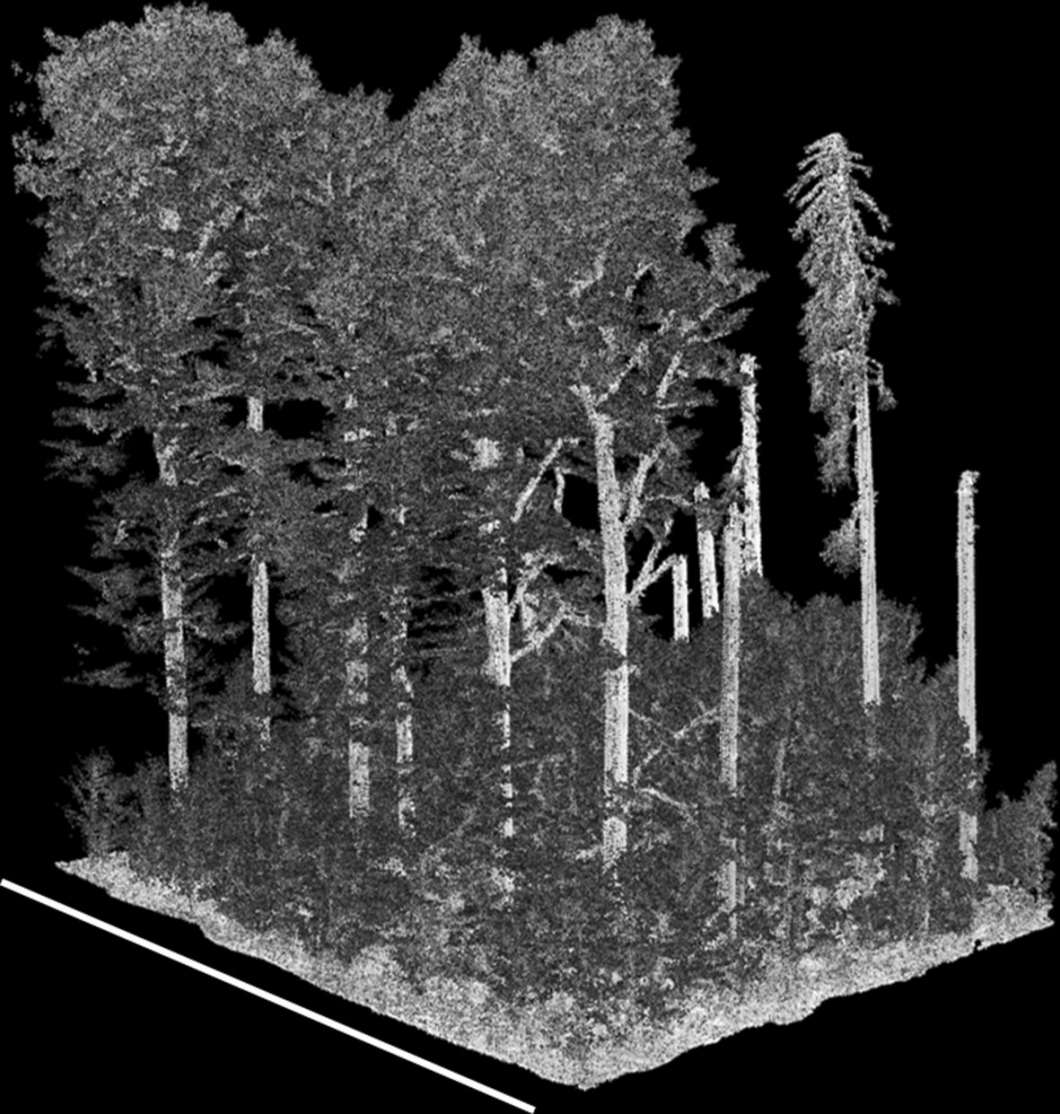
Stem and branch structure from high-density lidar acquired by a low-altitude drone. The point density in this scene is 3981 · m^−2^. Scale varies from this perspective. The length of the white bar is 30 m

**Fig. 7:**
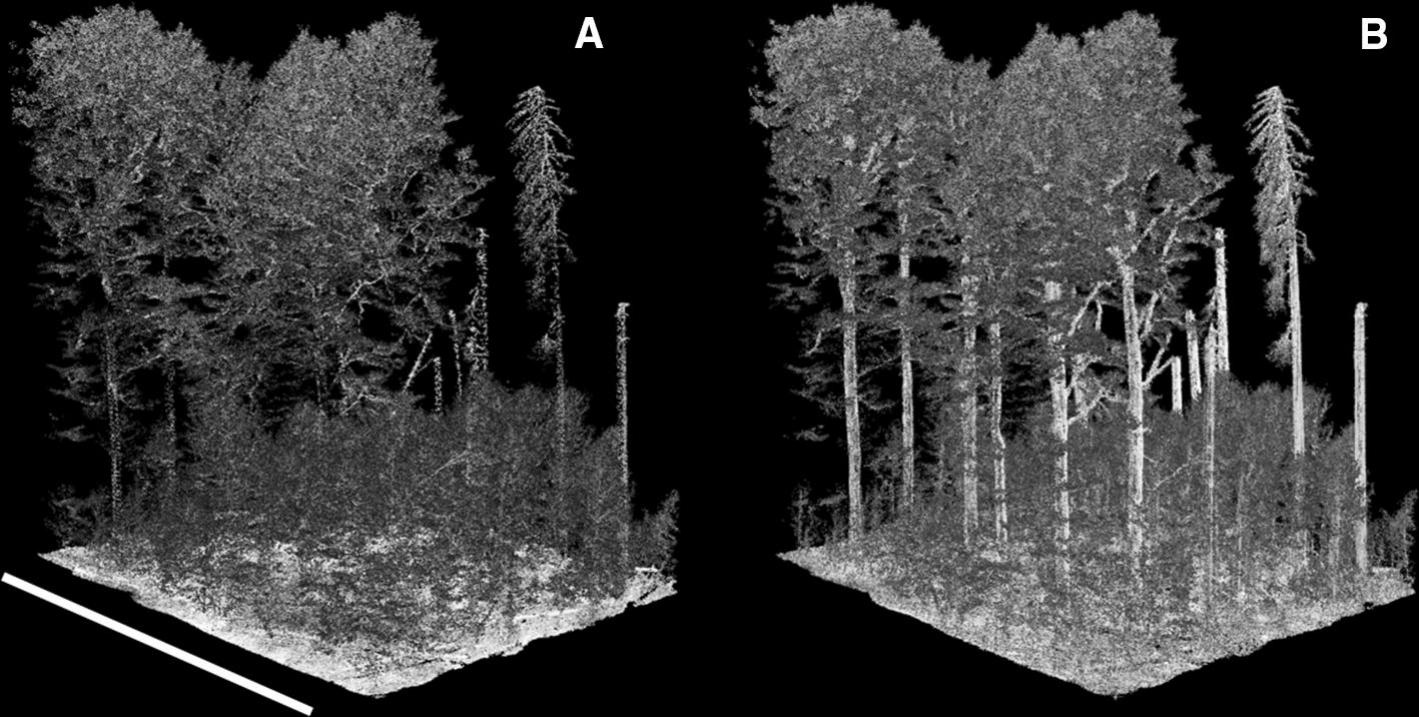
The impact of scan angle on measurements of stem and branch structure. The area is the same as Fig. 6. **a** Only points acquired from absolute scan angles < 10°. **b** Only points acquired from absolute scan angles > 30°. Most returns from stem and branch positions are from wide scan angles. Scale varies from this perspective. The length of the white bar is 30 m

The high-density point clouds collected in the southern Czech Republic resolve individual stem and branch structure (Figs. 5, 6). It is clear that measurements from wide scan angles are disproportionately important sources of information about stem structure, a finding also reported by Brede et al. (2017). This is apparent by examining the subset of old-growth forest in Fig. 7. The left panel contains only points from absolute scan angles < 10°, which is comparable to traditional airborne laser scanning, though much more densely sampled. The panel on the right shows the same location, but contains only points from absolute scan angles > 30°. Stem structure is more clearly resolved from measurements > 30° than from measurements < 10°. To determine the potential for automated stem segmentation using these data, we applied a low-intensity filter to remove non-wood returns. This indicates that branches are resolvable using measurements from low-altitude drone flight (Fig. 5).

### High-Frequency Observation

Because drone flight is more firmly under the control of the investigator and much less costly than traditional airborne laser scanning, flight plans can be developed to collect high-density measurements in novel ways that enable hypothesis testing or evaluate the impact of collection scenarios on remote sensing data. There is considerable debate over the role of leaf phenology, age, sensor and atmospheric artifacts in Amazon greening apparent in broadband satellite data (Saleska et al. 2007; Samanta et al. 2010; Morton et al. 2014; Tang and Dubayah 2017). High-frequency observations from drones could serve as an important source of information in this debate, for example, by observing short-term changes in leaf area and condition. The data we collected in the southern Czech Republic were designed in part to determine the impact of leaf area on simulated GEDI waveforms in the absence of changes in woody structure. By collecting measurements 51 days apart, we are able to observe changes associated with leaf flushing and phenology that are independent of woody structure.

Comparing measurements from leaf-off and leaf-on conditions in the Czech Republic demonstrates that the difference in point density is due to a reduction in the number of higher-order returns in the presence of leaf area (Table 2). For example, the number of first returns under leaf-off conditions in the 2.25 ha forested area was 41,822,033, which is a mean density of 1859 · m^−2^. Under leaf-on conditions, the number of first returns was 43,132,311 or 1917 · m^−2^. The total point density and distribution of return numbers for the 2.25 ha forested area can be contrasted with an open, recently disturbed location of 0.75 ha, some of which is illustrated in Fig. 3. This location contains 14,620,517 first returns under leaf-off conditions, and 17,879,244 first returns under leaf-on conditions, corresponding to densities of 1949 · m^−2^ and 2384 · m^−2^, respectively. The distribution of return numbers is more strongly skewed toward first returns under leaf-on conditions (Table 2). Another opportunity is observing short-term changes in condition, for example due to leaf wilting. These observations could be made by conducing repeated drone flights over diurnal time scales.

### Increasing the Representation of Ground-Based Field Inventories in Support of Mission Science

The Committee on Earth Observation Satellites (CEOS) is responsible for coordinating non-military Earth observations among national and international space agencies and other members (Duncanson et al. 2019). In concert with the Group on Earth Observations (GEO), the CEOS Working Group on Calibration and Validation (WGCV) Land Product Validation (LPV) subgroup has developed a forthcoming protocol focused on recommendations for validation of satellite AGBD data products. These recommendations include a call for increasing the number and quality of validation sites, highlighting the importance of permanent field plots (CEOS Strategy for Carbon Observations from Space 2014; CEOS Working Group on Calibration and Validation 2017). These recommendations recognize the critical role of ground-based field-inventory data in supporting the calibration and validation needs of current and forthcoming generations of land surface remote sensing.

Unfortunately, there are currently few locations in the world with large permanent-inventory plots suitable for calibration and validation of space mission data products. Even when field data exist, these inventories have usually not been professionally surveyed in an absolute coordinate system, making it difficult to align field data with airborne or satellite data products. Producing stem maps in large inventory plots is time-consuming and cannot be completed rapidly. These problems introduce a spatial and temporal mismatch between the field inventories and remotely sensed data (Réjou-Méchain et al. 2015). Other issues include changing points of measurement on individual tree stems over time due to the development of buttresses or other irregularities and inconsistent application of measurement protocols (Clark 2002; Metcalf et al. 2009).

All of these problems could be overcome by an operational framework to segment individual stems using ultra-high-density laser scanning from TLS and drones (Calders et al. 2015; Brede et al. 2017; Gonzalez de Tanago et al. 2018; Disney et al. 2018). Because the stem map can be derived directly from lidar data, spatial and temporal sources of uncertainty between field measurements and remote sensing are eliminated. Segmentation algorithms can be clearly described and placed in the public domain (Raumonen et al. 2013; Trochta et al. 2017). Uncertainties can be rigorously quantified and propagated, and algorithms can be applied consistently, ensuring that all data can be judged and evaluated by the same standard. This information could be rapidly generated using a coordinated campaign involving terrestrial and drone-based sensors over existing field inventories. For example, the Forest Global Earth Observatory plot network contains the largest permanent plots in the world, typically 25–50 ha in size. This network represents 66 sites in 27 countries, including 6 million individual trees and 10,000 species (Anderson-Teixeira et al. 2015).

Sampling these plots within regions of 1–10 km^2^ would provide the measurements necessary for calibration and validation of space mission data products. Acquisition of these data is possible using a heavy-lift or long-duration drone at a fraction of the cost of traditional airborne surveys. What would it take to accomplish this task? Using our experiences in the southern Czech Republic as a guide, generating 5000 points · m^−2^ requires about 5 flight hours · km^−2^. If lower point densities are acceptable, 50 points · m^−2^ can be achieved in about 6 min · km^−2^.

## Conclusions

Low-altitude flight using drones enables the collection of ultra-high-density point clouds using wider laser scan angles than have been possible from traditional airborne platforms. These measurements can be precise and accurate and can achieve measurement densities of thousands of points m^−2^. The 3D model produced by these data can clearly resolve branch and stem structure that is comparable to TLS and can be acquired rapidly over large landscapes at a fraction of the cost of traditional airborne laser scanning. Drone remote sensing is not a replacement for traditional airborne platforms or TLS. It is a tool for a different job. Drone-based flight operations provide flexibility and the potential to take advantage of unexpected opportunities. Drones may enable access to locations where traditional flight operations are challenging, either because sites are remote or because permissions are difficult to secure. The diversity of lightweight laser scanners and drone platforms creates opportunities to collect focused measurements of relatively small areas at low cost and the ability to characterize large landscapes that rival the extent of traditional airborne laser scanning. Measurements from drones are qualitatively distinct from traditional airborne laser scanning, owing to the large point densities and wide scan angles that are made possible by low-altitude flight.

Critical to global validation of the AGBD data products being produced by current and forthcoming space missions is access to globally representative samples of airborne lidar that characterize the diversity of relationships between vertical forest structure and AGBD. Drone acquisitions can contribute to producing these calibration and validation data sets.
